# The opposite roles of PAS-5 and Galectin-1 in immune response during the early infection of *Angiostrongylus cantonensis*

**DOI:** 10.1186/s13071-018-2894-5

**Published:** 2018-05-29

**Authors:** Lan-Zhu Yan, Xiao-Meng Shi, Yan-Wen Zu, Yuan-Yuan Shen, Xi-Xi Chen, Meng-Jing Zhao, Xing-Pan Li, Bao-Long Yan, Hui-Cong Huang

**Affiliations:** 1 Department of Parasitology, School of Basic Medical Sciences, Wenzhou Medical University, Wenzhou, Zhejiang 325035 People’s Republic of China; 2 Clinical Laboratory of Bethune International Peace Hospital, Shijiazhuang, Hebei 050082 People’s Republic of China; 3 School of the Second Clinical Medical Sciences, Wenzhou Medical University, Wenzhou, Zhejiang 325035 People’s Republic of China; 4 School of the First Clinical Medical Sciences, Wenzhou Medical University, Wenzhou, Zhejiang 325035 People’s Republic of China

**Keywords:** *Angiostrongylus cantonensis*, Early infection, PAS-5, Galectin-1, Immune response

## Abstract

**Background:**

*Angiostrongylus cantonensis* is a human zoonotic nematode parasite. Our previous studies found that PAS-5 and Galectin-1 (Gal-1) proteins of *A. cantonensis* could be strongly recognized by sera from mice infected with *A. cantonensis.* In this study, we further evaluated the potential roles of these two proteins in the induction of immune response in mice.

**Methods:**

Mice were immunized with recombinant PAS-5 or Gal-1 and then challenged with 30 infective *A. cantonensis* larvae following the last immunization. We then examined the infected mice for changes in serum antibodies and cytokines by ELISA, CD4^+^ T cells and CD4^+^CD25^+^FoxP3^+^ regulatory T cells (Tregs) by flow cytometry, and tissue damage severity by hematoxylin-eosin (H&E) staining.

**Results:**

Compared with control mice, the PAS-5-immunized mice exhibited increased levels of serum antibodies and cytokines (except for IL-10) at different time points post-infection. PAS-5 immunization promoted significant proliferation of CD4^+^ T cells, and caused more damage in the brain tissue. Vaccination with Gal-1 inhibited the production of antibodies (except for IgG1) and IFN-γ, but promoted the expression of IL-4 and IL-10. Gal-1 immunization results in significant increases in the levels of CD4^+^CD25^+^FoxP3^+^ Tregs, and mild inflammatory changes.

**Conclusions:**

Taken together, our findings show that PAS-5 enhances, but Gal-1 inhibits the immune response in the early stage of *A. cantonensis* infections.

## Background

*Angiostrongylus cantonensis*, also known as rat lungworm, is a parasitic nematode that can cause zoonotic infections [1, 2]. This species requires both intermediate host (slugs, snails and other mollusks) and definitive host (rats and other rodents) for completing the life-cycle. The first-stage larvae (L1) mainly exist in the feces of definitive hosts and develop into the infective third-stage larvae (L3) in the intermediate hosts. In definitive hosts, L3 migrate to the central nervous system (CNS) and develop into the fourth- and the fifth-stage larvae (L4 and L5). The L5 then migrate to the pulmonary artery and heart of the definitive hosts *via* the circulatory system, where they develop into adult nematodes [2–4]. Humans, despite being unsuitable hosts, can become infected by drinking/eating water/food contaminated by L3 of *A. cantonensis*, leading to eosinophilic meningitis, eosinophilic encephalitis [3–5], and ocular angiostrongyliasis [6]. *Angiostrongylus cantonensis* and angiostrongyliasis have been extensively studied, but the underlying pathogenic mechanisms are still largely unknown [7–9].

Previously, by utilizing two-dimensional gel electrophoresis (2-DIGE) and mass spectrometry (MS), we found that several interesting proteins of *A. cantonensis*, including Gal-1 and PAS-5, could be strongly recognized by sera from mice infected with *A. cantonensis* [3, 4, 10]. PAS-5 is α5 proteasomal subunit and plays a major role in inducing host immune response [11]. The proteasomes of parasites may alter the morphology of cells within the host, remove damaged and defunct proteins, and execute other functions to participate in the immune response [12]. Galectins are involved in various physiological and pathological processes, including RNA transcription, cell adhesion, cell apoptosis and immune regulation [3, 4, 13]. Of all galectin members, the most studied is Gal-1, which has been found to be involved in the regulation of the immune response [14]. We here evaluate the effects of PAS-5 and Gal-1 on host immune response in the early stage of *A. cantonensis* infection. We screened the infected mice for changes in serum antibodies, cytokines, and CD4^+^ T cells and CD4^+^CD25^+^FoxP3^+^ Tregs, as well as for differences in tissue damage severity. The results of our study provide insight into the pathogenic mechanism of this organism, and could comprise a theoretical basis for treating angiostrongyliasis.

## Methods

### Gene cloning and expression

The total RNA of *A. cantonensis* was extracted using Trizol reagent and reversely transcribed into cDNA. The full-length open reading frames (ORFs) of PAS-5 and Gal-1 were amplified by PCR using following primer pairs. PAS-5: PAS-5-Forward, 5'-GT CATATG TTT CTA ACG CGA AGT G-3', and PAS-5-Reverse, 5'-CG TCTAGA **ATG ATG ATG ATG ATG ATG** CAA ACT TGA AAT GAC AAC G-3'; Gal-1: Gal-1-Forward, 5'-CG GGAT CC **CAT CAT CAT CAT CAT CAT** ATG TCG TCT CCT CCA-3', and Gal-1-Reverse, 5'-CT TCTAGA CTA CTG AAT TTG AAT GCC GGT-3'. The 6xHis tag-encoding sequences are marked in bold. The ORFs of PAS-5 and Gal-1 were then subcloned into pColdIII to generate plasmids pColdIII-PAS-5 and pColdIII-Gal-1, respectively. The resulted plasmids were transformed into *E. coli* BL21, and the recombinant proteins (rPAS-5 and rGal-1) were expressed by IPTG induction and purified with NI-NTA beads.

### Animals and parasites

*Pomacea canaliculata*, the intermediate host of *A. cantonensis*, was supplied by a peasant household on a private property in Cangnan County, Wenzhou City, Zhejiang Province, China, and consent was obtained from this household to use the intermediate host for our study. Upon receipt, several *P. canaliculata* snails were selected at random for dissection and microscopic analysis to ensure that they were not infected with *A. cantonensis* or any other species of worm. After this confirmatory analysis, the remaining snails were simultaneously infected with L1 of *A. cantonensis* to ensure that all resulting L3 were of the same origin. The C57BL/6 female mice (5–6 weeks-old, grade SPF) were supplied by the Laboratory Animal Center of Wenzhou Medical University (Zhejiang, China). Laboratory reared Sprague-Dawley rats that were infected with L3. The L3 were obtained as previously described [3, 4].

### Immunity and infection

Seventy-two mice were randomly and equally divided into three groups: group 1, mice immunized with rPAS-5 plus adjuvant; group 2, mice immunized with rGal-1 plus adjuvant; and group 3, mice immunized with PBS plus adjuvant. The mice were immunized using the procedure shown in Table 1. For the first round of immunization, Freund’s complete adjuvant (Sigma-Aldrich, St. Louis, MO, USA) was used, while Freund’s incomplete adjuvant (Sigma-Aldrich) was used for the second and the third rounds. The recombinant protein plus adjuvant compound was emulsified by pumping it through a syringe repeatedly until a “water-in-oil” state was achieved. The immunization was performed thrice at one-week intervals.

**Table 1:** Experimental protocol: procedure for immunization of mice against *A. cantonensis*

Day	Injection	Group 1	Group 2	Group 3
0	First	100 μg rPAS-5/mouse + adjuvant	100 μg rGal-1/mouse + adjuvant	PBS + adjuvant
7	Second	50 μg rPAS-5/mouse + adjuvant	50 μg rGal-1/mouse + adjuvant	PBS + adjuvant
14	Third	50 μg rPAS-5/mouse + adjuvant	50 μg rGal-1/mouse + adjuvant	PBS + adjuvant
21	Sacrifice and collect the samples (0 weeks before infection)			
Challenge with 30 *A. cantonensis* larvae/mouse				
28	Sacrifice and collect the samples (1 week after infection)			
35	Sacrifice and collect the samples (2 weeks after infection)			
42	Sacrifice and collect the samples (3 weeks after infection)			

One week after the last immunization, mice were challenged with 30 L3 by oral inoculation, and then screened for changes in serum antibodies, cytokines, and CD4^+^ T cells and CD4^+^CD25^+^FoxP3^+^ Tregs, and for tissue damage severity at weeks 0 (before infection), 1, 2 and 3.

### Evaluation of humoral immune responses by ELISA

Serum levels of total IgG, IgG1, IgG2a and IgE were determined at different time points by ELISA analysis (eBioscience, San Diego, CA, USA) according to the manufacturer’s instructions. Briefly, microtiter plates were coated with capture antibodies diluted in coating buffer (1:250) overnight at 4 °C, and blocked with 200 μl blocking buffer at 37 °C for 2 h. Then, 100 μl of serum or standards were added, followed by 50 μl of HRP-conjugated detection antibody (1:250). After incubation for 3 h, 100 μl of tetramethylbenzidine substrate solution was added, and the reaction was subsequently stopped with addition of 100 μl of stop solution. Lastly, the absorbance of each well at 450 nm was measured using a plate reader, a standard curve was established according to the OD values, and antibody concentrations were calculated. All samples were assayed in triplicate.

### Cytokine measurements

To observe the influence of PAS-5 and Gal-1 on cellular immunity, we measured the levels of cytokine production splenic cells upon treatment with each protein *in vitro*. First, the spleens of immunized and control mice were removed. Splenic cells were then harvested by grinding of the splenic tissues, centrifuging, RBC cracking, and centrifuging again. The resulting cell pellets were suspended in Dulbecco’s modified Eagle’s medium (DMEM) (Gibco, Grand Island, NY, USA) and filtered with strainer mesh. Aliquots of the suspensions were then subjected to cultivation (1 × 10^6^ cells/ml) and flow cytometry analysis (1.5 × 10^7^ cells/ml). Splenic cells cultivated in 96-well plates were stimulated with PAS-5 (100 ng/ml) or Gal-1 (25 ng/ml) at 37 °C for 72 h in a humidified 5% CO_2_ atmosphere. Supernatants were then collected, and the levels of IFN-γ, IL-4, IL-5 and IL-10 were measured by ELISA analysis (BD Biosciences, San Diego, CA, USA). Cells stimulated with concanavalin A (ConA, Sigma-Aldrich) and PBS were used as positive and negative controls, respectively. The absorbance of each plate was read at 450 nm, and a standard curve was established. All samples were assayed in triplicate.

### Flow cytometry

Splenic cells were blocked with FACS blocking buffer for 30 min, then incubated with anti-CD4-FITC, and anti-CD25-APC antibodies (BD Biosciences) for 30 min on ice. Samples were treated with fixation/permeabilization buffer, and permeabilized cells were stained using anti-FoxP3-PE antibodies (BD Biosciences) for 30 min on ice. Data were acquired using a FACS flow cytometer and analyzed with FlowJo software.

### Histopathological observation

Specimens of brain tissue were fixed in 4% paraformaldehyde for 2 days, dehydrated with a graded series of ethanol, embedded in paraffin, sectioned, stripped, and stained with hematoxylin-eosin (H&E). Specimens were then subjected to microscopic analysis to evaluate the levels of inflammatory cell infiltration and pathological changes and compare degrees of inflammation and damage between groups.

### Statistical analysis

Data are presented as means ± standard errors of the mean (SEM). The comparisons among 3 groups were performed using randomized block-designed ANOVA. If the difference reached a significant level, a *post-hoc* test was applied to investigate. All analyses were performed with SPSS 20.0 software (SPSS Statistics, Inc., Chicago, IL, USA). *P* < 0.05 was considered statistically significant.

## Results

### PAS-5 and Gal-1 modulate the humoral immune response

**Fig. 1:**
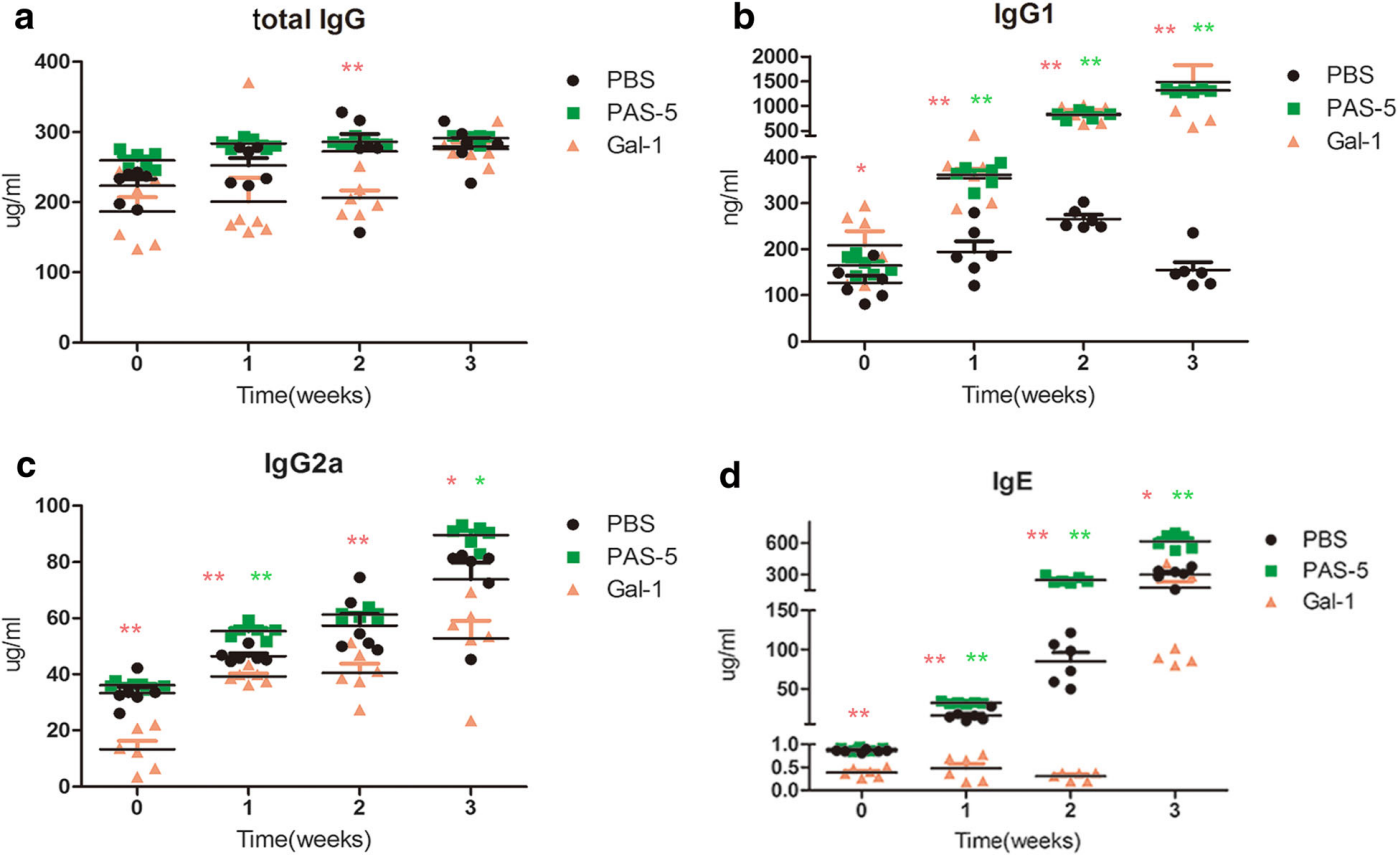
Changes in the levels of serum antibodies at different time points after infection with *A. cantonensis*. Graphic depiction of the levels of total IgG (**a**), IgG1 (**b**), IgG2a (**c**) and IgE (**d**) within the serum of control mice and mice treated with rPAS-5 or rGal-1, as determined by ELISA, represented as means ± SEM. The statistical analyses were performed using one-way ANOVA assays with *post-hoc* testing (*P < 0.05, **P < 0.01)

Compared with the control group at different times, mice treated with PAS-5 and Gal-1 respectively exhibited significantly increased serum levels of IgG1 (*F*_(2, 66)_ = 19.003, *P* < 0.0001; *F*_(3, 66)_ = 21.917, *P* < 0.0001, respectively) (Fig. 1b). Treatment with PAS-5 also exhibited significantly increased serum levels of IgG2a and IgE, while Gal-1 inhibited the generation of IgG2a (*F*_(2, 66)_ = 51.844, *P* < 0.0001; *F*_(3, 66)_ = 86.063, *P* < 0.0001, respectively), and IgE (*F*_(2, 66)_ = 24.143, *P* < 0.0001; *F*_(3, 66)_ = 58.921, *P* < 0.0001, respectively) (Fig. 1c-d). While compared with the control group, treatment with Gal-1 did not cause much difference in the levels of total IgG, except for a reduction at 2 weeks post-treatment (*F*_(2, 66)_ = 16.868, *P* < 0.0001; *F*_(3, 66)_ = 7.477, *P* < 0.0001, respectively) (Fig. 1a).

### PAS-5 and Gal-1 influence the cellular immune response

**Fig. 2:**
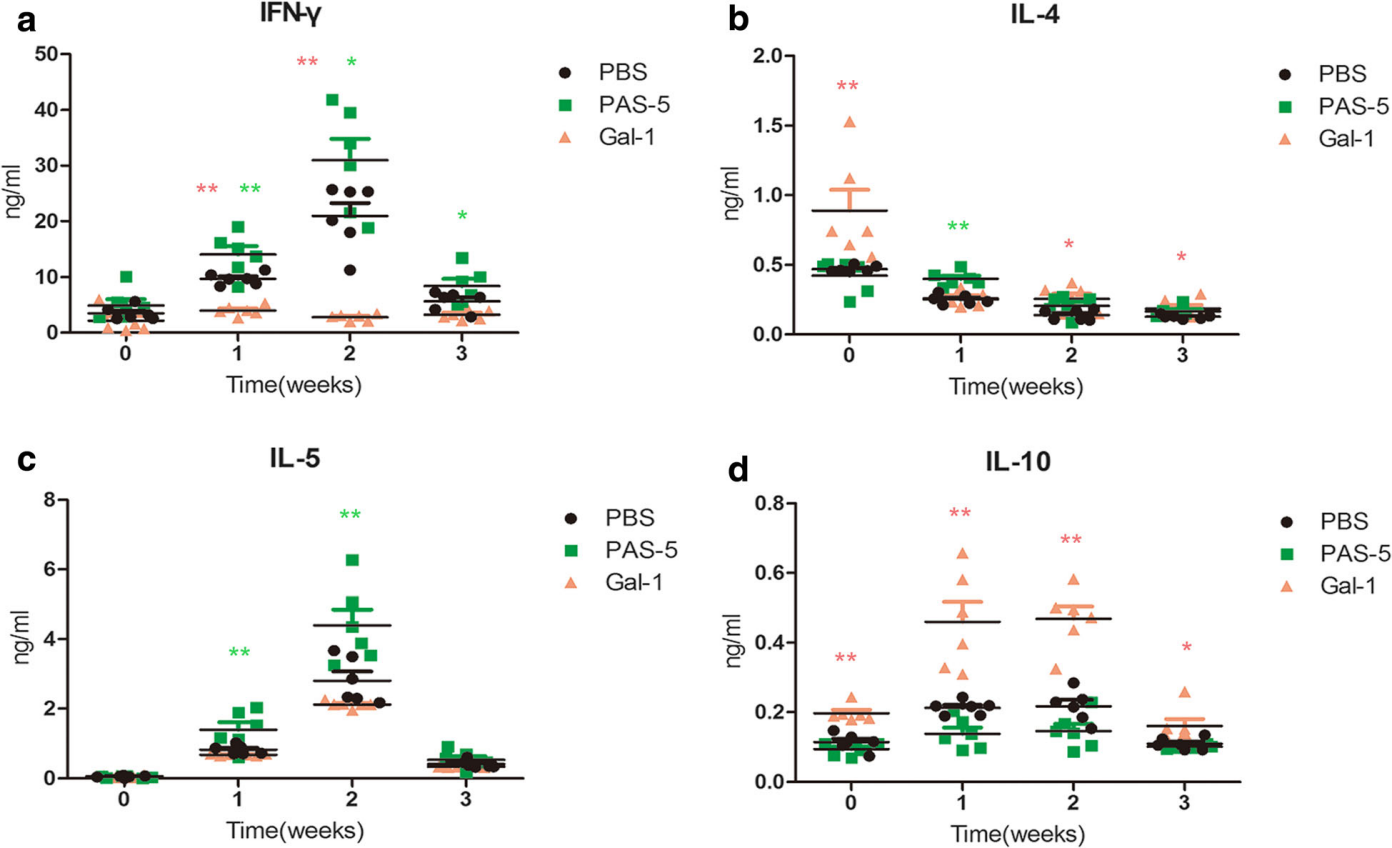
Changes in cytokine levels at different time points after infection with *A. cantonensis*, as measured by ELISA. Graphic depiction of the levels of IFN-γ (**a**), IL-4 (**b**), IL-5 (**c**) and IL-10 (**d**) within the cell supernatant of control mice and mice treated with rPAS-5 and rGal-1, as determined by ELISA, represented as means ± SEM. The statistical analyses were performed using one-way ANOVA assays with *post*-*hoc* testing (*P < 0.05, **P < 0.01)

After L3 infection, PAS-5-stimulated splenic cells produced high levels of IFN-γ, and cells treated with Gal-1 exhibited low levels (*F*_(2, 66)_ = 25.944, *P* < 0.0001; *F*_(3, 66)_ = 24.175, *P* < 0.0001, respectively) (Fig. 2a). Compared with the control group, cells treated with Gal-1 resulted in an increase in the levels of IL-4 at all chase time periods, while in PAS-5-treated cells, the levels of IL-4 was increased only at 1 week post-treatment (*F*_(2, 66)_ = 5.930, *P* = 0.004; *F*_(3, 66)_ = 29.769, *P* < 0.0001, respectively) (Fig. 2b). PAS-5-treated cells produced high levels of IL-5, and cells treated with Gal-1 exhibited lower levels than PAS-5, but had no significant difference compared with the control (*F*_(2, 66)_ = 12.828, *P* < 0.0001; *F*_(3, 66)_ = 107.637, *P* < 0.0001, respectively) (Fig. 2c). Meanwhile, the level of IL-10 was high in the Gal-1 group (*F*_(2, 66)_ = 46.622, *P* < 0.0001; *F*_(3, 66)_ = 21.660, *P* < 0.0001, respectively) (Fig. 2d).

### PAS-5 and Gal-1 modulate the immune response *via* CD4^+^T and CD4^+^CD25^+^FoxP3^+^ Tregs

**Fig. 3:**
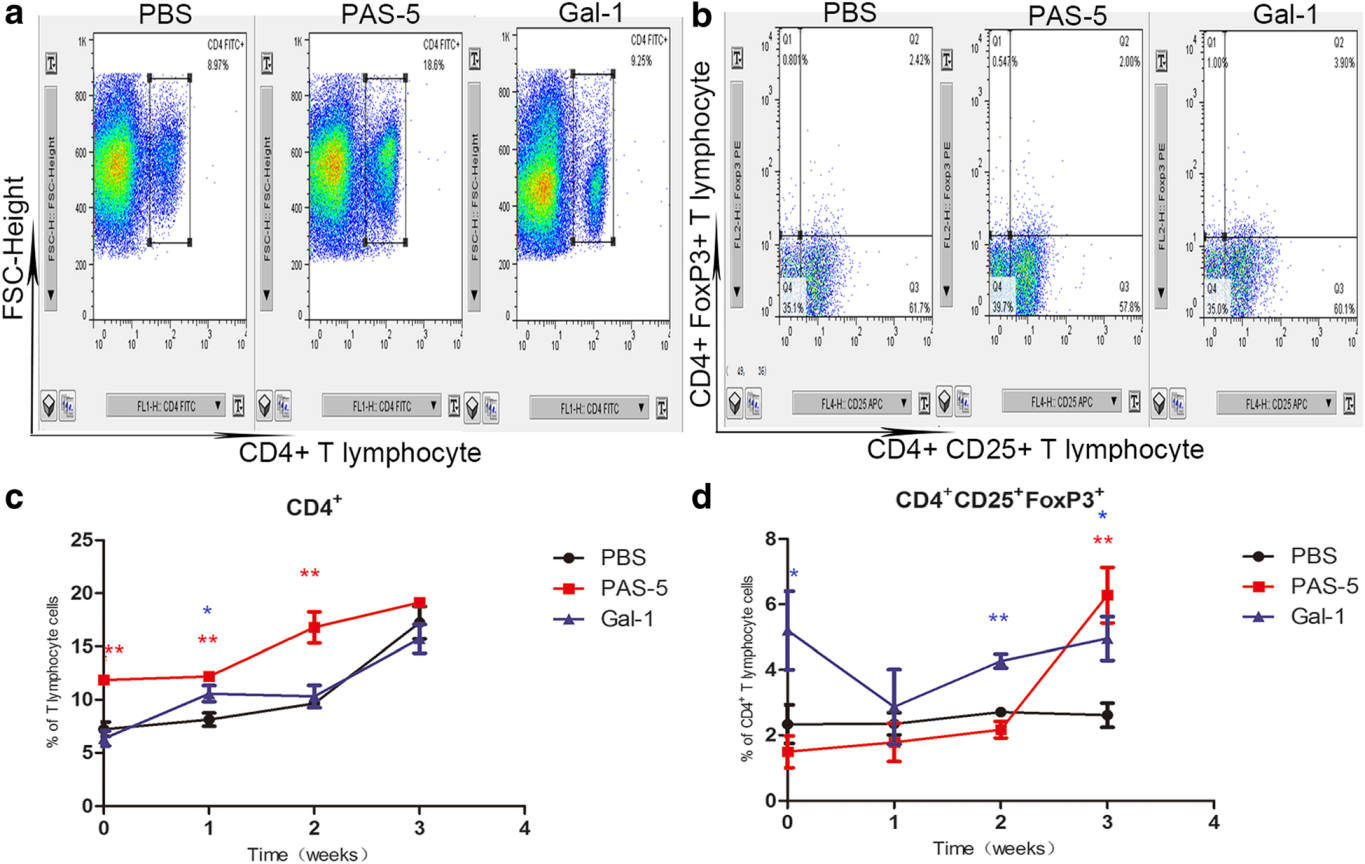
Changes in the populations of immune effector cells at different time points after infection with *A. cantonensis*. **a**, **b** Graphic depictions of the percentages of CD4^+^ T cells (**a**) and CD4^+^CD25^+^FoxP3^+^ Tregs (**b**) within the cells of control mice and mice treated with rPAS-5 and rGal-1 at 2 weeks after infection. **c**, **d** Graphic depictions of changes in the populations of CD4^+^ T cells (**c**) and CD4^+^CD25^+^FoxP3^+^ Tregs (**d**) at different time points after L3 infection, represented as means ± SEM. The statistical analyses were performed using one-way ANOVA assays with *post-hoc* testing (*P < 0.05, **P < 0.01)

After infection with L3, there was a progressive increase in the population of CD4^+^ T cells treated with PAS-5 (Fig. 3c). There was no significant distinction in spite of a progressive decrease in the number of CD4^+^CD25^+^FoxP3^+^ Tregs 2 weeks after infection with L3 among the mice treated with PAS-5 (*P* > 0.05), while it was increased at 3 weeks compared with those of the control (Fig. 3d). Meanwhile, there was a significant increase in the population of CD4^+^CD25^+^FoxP3^+^ Tregs (Fig. 3d), and almost nothing had changed in the number of CD4^+^ T cells among the mice in the Gal-1 group (*P* > 0.05) (Fig. 3c).

### Histopathological changes

**Fig. 4:**
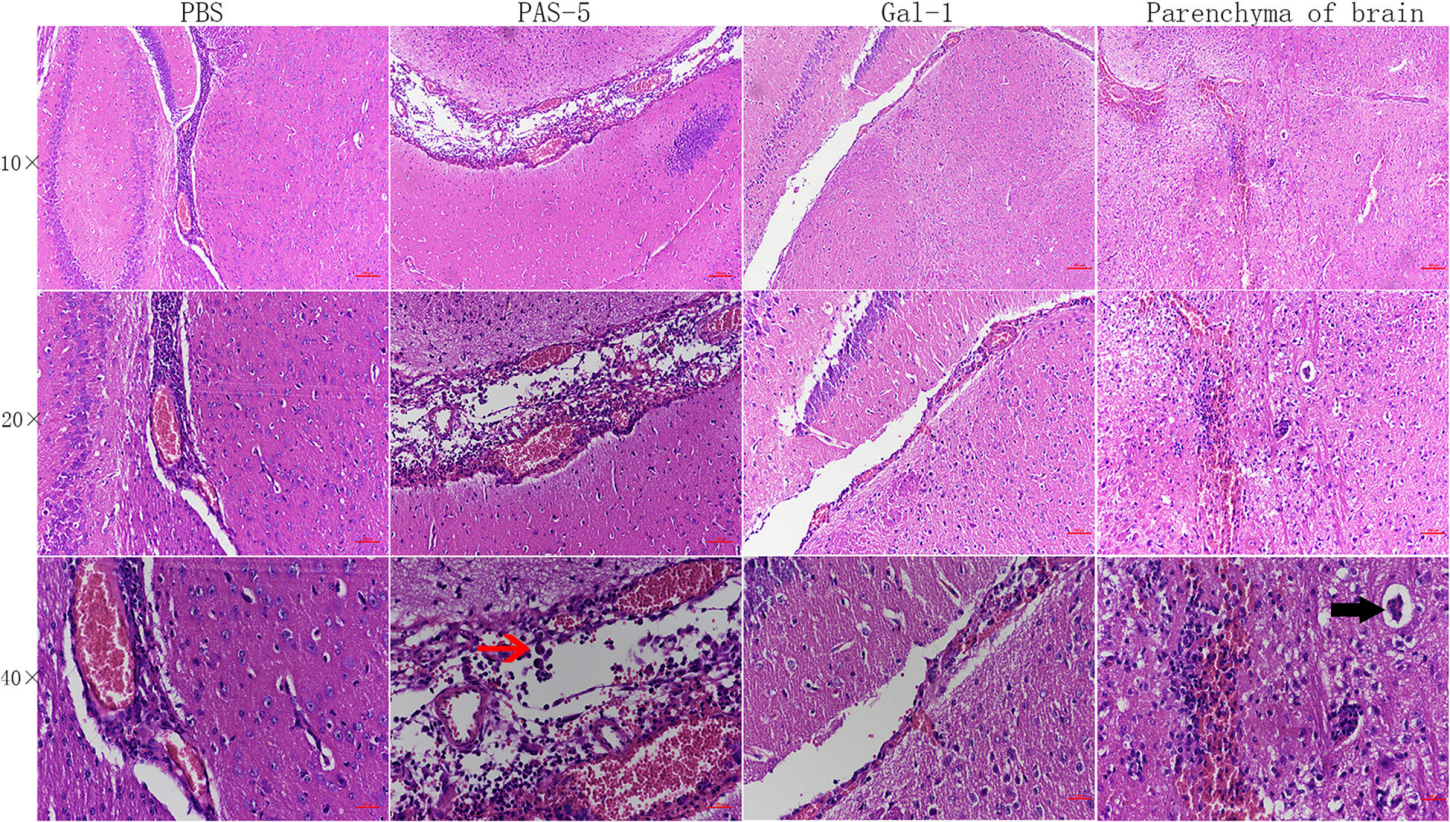
Histopathological changes in the brains of mice at 3 weeks after *A. cantonensis* infection. The red arrow represents eosinophil infiltration, and the bold arrow represents perivascular cuffing in the parenchyma of the brain. *Scale-bars*: 100 μm

Histopathological examination of the brains of the mice in control group detected no change at 1 week after infection, the presence of small numbers of inflammatory cells at week 2, and expansion and hyperemia within the brain capillary, and the infiltration of large numbers of inflammatory cells at week 3 (Fig. 4). Meanwhile, only mild inflammatory changes and infiltration of small numbers of inflammatory cells were observed in Gal-1-treated mice at 2 weeks and 3 weeks post-infection, respectively (Fig. 4). Conversely, serious tissue damage was observed in the brains of PAS-5-treated mice. Specifically, some inflammatory cell infiltrating was observed 1 week after infection; subsequently, progressively larger numbers of neutrophils, lymphocytes and eosinophils were observed at 2 and 3 weeks post-infection (Fig. 4). Concurrently, the parenchyma of the brains of these mice exhibited capillary expansion, as well as infiltration of lymphocytes to the area surrounding these vessels, resulting in perivascular cuffing (Fig. 4).

## Discussion

Although considerable research has focused on the role of proteasomal PAS-5 and Gal-1 in cancer and inflammation [15–19], the effects of these proteins on parasitic infections that affect the immune system, particularly on *A. cantonensis* infection, are not well understood*.* Mice and humans are both non-permissive hosts of *A. cantonensis*, and the CNS symptoms associated with angiostrongyliasis are similar [6]. Thus, mice are typically selected as appropriate animal models for studying *A. cantonensis* infection. Because angiostrongyliasis is associated with acute inflammation, we hence examined the immune responses to Gal-1 and PAS-5 within the first three weeks after infection. IgG is well known as a key component in humoral immune response. Moreover, the IgG subtypes IgG1 and IgG2a respectively indicate the Th2 and Th1 response and the levels of IgE reflect the changes of eosinophils. We thus detected these immunoglobulins here. We found that PAS-5 induced, but Gal-1 inhibited, the humoral immune response. In particular, PAS-5 triggered both the Th1 and Th2 response, while Gal-1 inhibited the Th1 and induced the Th2 response. Previous studies showed that *A. cantonensis* infection can lead to high levels of eosinophils in both the peripheral blood and cerebrospinal fluid [3], and that eosinophils were capable of killing *A. cantonensis* larvae on infected mice by discharging granules [9, 20–22]. In the present study, treatment of mice with PAS-5 and Gal-1 stimulated and inhibited IgE production, respectively, thereby strengthening and weakening the host immune response after L3 infection.

IFN-γ is a crucial cytokine for Th1 polarization [23], and our results show that *A. cantonensis* infection led to continuous increase levels of IFN-γ until three weeks post-infection, suggesting a transfer to the Th2-type immune response through Th1. IL-4 is primarily produced by Th2 cells [24, 25], and the production of IFN-γ inhibits the activation and proliferation of Th2. We observed low levels of IL-4 production during the early stage of *A. cantonensis* infection. Meanwhile, IL-5 plays an important role in the regulation of eosinophil formation, maturation, recruitment and survival [9, 20, 26], and IL-10 could promote the down-regulation of co-stimulatory surface molecules of macrophages and inhibition of cellular proliferation, which is beneficial to parasite survival [27]. Previously, Gal-1 combined with a heavily glycosylated isoform of CD45 on the surface of undifferentiated activated Th cells was shown to facilitate the development of a molecular circuit that enhances IL-10 production and suppresses the immune response [16]. We here found that PAS-5 promoted IFN-γ and IL-4 production, induced high levels of IL-5. Conversely, Gal-1 inhibited IFN-γ secretion, but promoted IL-4 and IL-10 production. Thus, similar to our observations regarding the humoral immune response, PAS-5 and Gal-1 also induced and inhibited the cellular immune response, respectively.

PAS-5 and Gal-1 modulate the immune response *via* CD4^+^ T cells and CD4^+^CD25^+^FoxP3^+^ Tregs. T cells are comprised of two major subgroups: CD4^+^ helper cells and CD8^+^ cytotoxic cells. Notably, studies have shown that CD4^+^ T cells play a primary role in the early infection of *A. cantonensis* [28]*.* Our results showed that PAS-5 significantly promoted the production of CD4^+^ T cells after L3 infection, while Gal-1 had little effect on this cell type, compared with the control group. Meanwhile, CD4^+^CD25^+^FoxP3^+^ Tregs play an important role in parasite-mediated down-regulation of the immune system [28, 29], and most helminth infections result in recruitment of Tregs [30]. Two different mechanisms have been proposed for the inhibition of Tregs during parasitic infection. The interplay between the T effector ligands CD80 and CD86 with cytotoxic T lymphocyte-associated protein-4 activates the transfer of immunosuppressive signals on effector T cells, thereby reducing the function of the latter [29]. Additionally, Tregs secrete IL-10 and TGF-β, which mediate immunosuppression [24, 30–32]. Consistent with our previous results, PAS-5 and Gal-1 exhibited immunoenhancing and immunosuppressing effects by inhibiting and promoting Treg secretion, respectively. It is noteworthy that PAS-5 induced high levels of Tregs at week 3 post-infection, as eosinophils were shown to release granules, as well as certain cytotoxic proteins, such as eosinophil cationic protein and eosinophil protein X, during this time period for killing the larvae [9, 22, 30]. The release of these factors induces nerve tissue damage within the host, after which the immune response is suppressed.

H&E staining of the brain tissues of infected mice further verified the effects of PAS-5 and Gal-1 on the CNS. The infection of *A. cantonensis* mainly damages the ventricles of the brain and the subarachnoid cavity, and a strong immune response will contribute to the severe damage of the parenchyma of the brain [9, 30]. Concordantly, we observed that PAS-5 treatment resulted in severe damage to the brain, including the parenchyma, while Gal-1 caused only slight damage, compared with the control group.

## Conclusions

In summary, our findings revealed that PAS-5 and Gal-1 have distinct effects on the induction of the immune system of mice infected with *A. cantonensis.* PAS-5 enhances both the humoral and cellular immune response through the Th1 and Th2, whereas Gal-1 inhibits the response through the Th1 pathway.

## Abbreviations

2-DIGE: Two-dimensional gel electrophoresis
CNS: Central nervous system
ELISA: Enzyme-linked immunosorbent assay
FACS: Fluorescene-activated cell sorting
H&E: Hematoxylin-eosin
L1: First-stage larvae
L3: Third-stage larvae
L4: Fourth-stage larvae
L5: Fifth-stage larvae
MS: Mass spectrometry
ORFs: Open reading frames
Tregs: Regulatory T cells
